# Detection of knee synovitis using non-contrast-enhanced qDESS compared with contrast-enhanced MRI

**DOI:** 10.1186/s13075-021-02436-8

**Published:** 2021-02-13

**Authors:** Bas A. de Vries, Stephan J. Breda, Bragi Sveinsson, Emily J. McWalter, Duncan E. Meuffels, Gabriel P. Krestin, Brian A. Hargreaves, Garry E. Gold, Edwin H. G. Oei

**Affiliations:** 1 Department of Radiology & Nuclear Medicine, Erasmus MC, Doctor Molewaterplein 40, 3015 GD Rotterdam, The Netherlands; 2 Department of Orthopedic Surgery, Erasmus MC, Rotterdam, The Netherlands; 3 Department of Radiology, Massachusetts General Hospital, Boston, USA; 4 Harvard Medical School, Boston, USA; 5 Department of Mechanical Engineering, University of Saskatchewan, Saskatoon, Canada; 6 Department of Radiology, Stanford University, Stanford, USA

**Keywords:** Osteoarthritis, Knee, Synovitis, Magnetic resonance imaging, Inflammation, Humans

## Abstract

**Background:**

To assess diagnostic accuracy of quantitative double-echo in steady-state (qDESS) MRI for detecting synovitis in knee osteoarthritis (OA).

**Methods:**

Patients with different degrees of radiographic knee OA were included prospectively. All underwent MRI with both qDESS and contrast-enhanced T1-weighted magnetic resonance imaging (CE-MRI). A linear combination of the two qDESS images can be used to create an image that displays contrast between synovium and the synovial fluid. Synovitis on both qDESS and CE-MRI was assessed semi-quantitatively, using a whole-knee synovitis sum score, indicating no/equivocal, mild, moderate, and severe synovitis. The correlation between sum scores of qDESS and CE-MRI (reference standard) was determined using Spearman’s rank correlation coefficient and intraclass correlation coefficient for absolute agreement. Receiver operating characteristic analysis was performed to assess the diagnostic performance of qDESS for detecting different degrees of synovitis, with CE-MRI as reference standard.

**Results:**

In the 31 patients included, very strong correlation was found between synovitis sum scores on qDESS and CE-MRI (*ρ* = 0.96, *p* < 0.001), with high absolute agreement (0.84 (95%CI 0.14–0.95)). Mean sum score (SD) values on qDESS 5.16 (3.75) were lower than on CE-MRI 7.13 (4.66), indicating systematically underestimated synovitis severity on qDESS. For detecting mild synovitis or higher, high sensitivity and specificity were found for qDESS (1.00 (95%CI 0.80–1.00) and 0.909 (0.571–1.00), respectively). For detecting moderate synovitis or higher, sensitivity and specificity were good (0.727 (95%CI 0.393–0.927) and 1.00 (0.800–1.00), respectively).

**Conclusion:**

qDESS MRI is able to, however with an underestimation, detect synovitis in patients with knee OA.

## Key messages

- qDESS synovitis images can differentiate between the synovial membrane and joint effusion.
- qDESS MRI is able to, with an underestimation, detect synovitis in patients with knee OA.

## Background

Osteoarthritis (OA) is the most common joint disease. In men and women over 60 years, 10% and 13% respectively suffer from symptomatic knee OA [1]. Joint inflammation, characterized by swelling of the synovium and joint effusion, is believed to be a key process of knee OA in half of all OA patients [2]. Synovial inflammation, also referred to as synovitis, already occurs in early OA [3] and plays an important role in OA symptom perception, with odds ratios (ORs) varying between 3.2 and 10.0 for effusion/synovitis [4, 5]. Pain is the most prevalent symptom of OA and is associated with inflammation [5]. Synovitis is also an important predictor of OA progression [6]. Hence, synovitis is considered a potential tissue-specific target for novel anti-inflammatory treatments [7]; In addition, synovitis has been suggested as a predictive factor of knee OA progression in worsening of cartilage damage, with accompanying ORs up to 3.11 for progression of pain on a visual analog scale (VAS) after 1 year [8]. As the prominent role of synovitis in OA is increasingly recognized, there is growing interest in identifying OA patients with synovitis by means of imaging for the purpose of personalized prognostication and therapy.

The most common method to image OA in routine patient care and large clinical studies consists of radiography, but this primarily only visualizes bony structures and cannot assess synovitis. Magnetic resonance imaging (MRI) is a very suitable method for imaging OA, because it offers a comprehensive assessment of multiple joint tissues involved in OA [9], including direct visualization of articular cartilage, subchondral bone, menisci, ligaments, and joint effusion as a surrogate marker of inflammation. Furthermore, MRI can directly visualize synovitis when an intravenous contrast agent is administered, also referred to as contrast-enhanced MRI (CE-MRI) [10]. CE-MRI is currently considered the reference standard for imaging of synovitis, because the direct visualization of thickened synovium is preferred over the assessment of joint effusion and these findings should be treated as two separate entities [11]. Thus, MRI complemented with CE-MRI is an excellent technique to study relationships between synovitis and other OA manifestations. However, because of high costs, longer examination times, and potential health risks associated with the intravenous contrast agent or undergoing repeated examinations, especially in patients with renal insufficiency and allergies, there is reluctance to implement synovitis imaging with CE-MRI in routine clinical MRI protocols and large clinical research studies [12]. These disadvantages of CE-MRI highlight the need for an imaging technique without the use of a contrast agent.

A promising recent innovation in MRI of synovitis is diffusion-weighted imaging with quantitative double-echo in steady-state (qDESS) MRI without the need for a contrast agent, which has higher resolution than conventional diffusion-weighted techniques, without off-resonance-induced distortion. qDESS is a 3D gradient-spoiled steady-state sequence, acquiring an echo before and after a spoiler gradient, which are usually combined to one image in the qDESS and used in the Osteoarthritis Initiative. The advantage of qDESS is that next to the diffusion image it can also be used to get a comprehensive image of an OA knee within 5 min [13]. In the 1980s, several groups [14–16] showed that the different contrasts of the two echoes are useful, and this was more recently demonstrated by Welsch et al. [17]. Further modification to [18, 19] qDESS by increasing the magnitude of the spoiler gradient between the two echoes and acquiring separate echoes, synovitis can be detected without the need for an intravenous contrast agent, as shown previously by McWalter et al. [20]. The images have different levels of diffusion weighting, enabling good separation of fluid and surrounding tissues. This work demonstrates the feasibility of visualizing synovitis using qDESS MRI [20]; specifically that qDESS MRI correlates well with CE-MRI in patients with moderate to advanced clinical synovitis.

Therefore, the purpose of this study was to assess the diagnostic performance of qDESS MRI for the assessment of knee synovitis in patients with a varying degree of radiographic knee OA, using CE-MRI as the reference standard. Based on our pilot study, we hypothesized that qDESS MRI has high diagnostic performance and that the addition of qDESS MRI to clinical scan protocols can be feasibly implemented on a larger scale in prospective clinical studies, in order to assess the prognostic value of synovitis and the response to interventions.

## Methods

### Study population

Patients with knee OA were included consecutively from the outpatient clinic of the Department of Orthopedic Surgery. The institutional review board approved the study, and informed consent was obtained from all subjects. Patients included for this study were aged over 18 years, with a severity of at least Kellgren and Lawrence (K&L) [21] grade 1 and had clinical suspicion of synovitis based on palpable joint effusion. Exclusion criteria were as follows: previous knee replacement surgery, knee trauma in the preceding 6 months, absolute and relative contra-indications to undergo MRI, pregnancy, renal insufficiency (GFR < 60 mL/min/1.73 m^2^), and a known allergy to MR gadolinium containing contrast agents.

### MR image acquisition

A 3-T MR system (Discovery MR750, General Electric Healthcare, Milwaukee, WI, USA) was used with a dedicated 8-channel knee coil (Invivo, Gainesville, FL, USA). For CE-MRI, we applied a sagittal 3D T1-weighted spoiled gradient-echo sequence (SPGR) with fat saturation obtained after the intravenous administration of 0.2 mmol/kg of gadoterate meglumine (Dotarem®, Guerbet, Aulnay-sous-Bois, France). The T1-weighted scan was performed 6 min after the intravenous administration of the contrast agent. Scan parameters of the T1-weighted scan were TR/TE = 10.8/5.4 ms, flip angle = 20°, FOV = 20 × 20 cm, slice thickness = 0.5 mm, matrix = 512 × 512, and receiver bandwidth = ± 62.5 kHz.

**Fig. 1:**
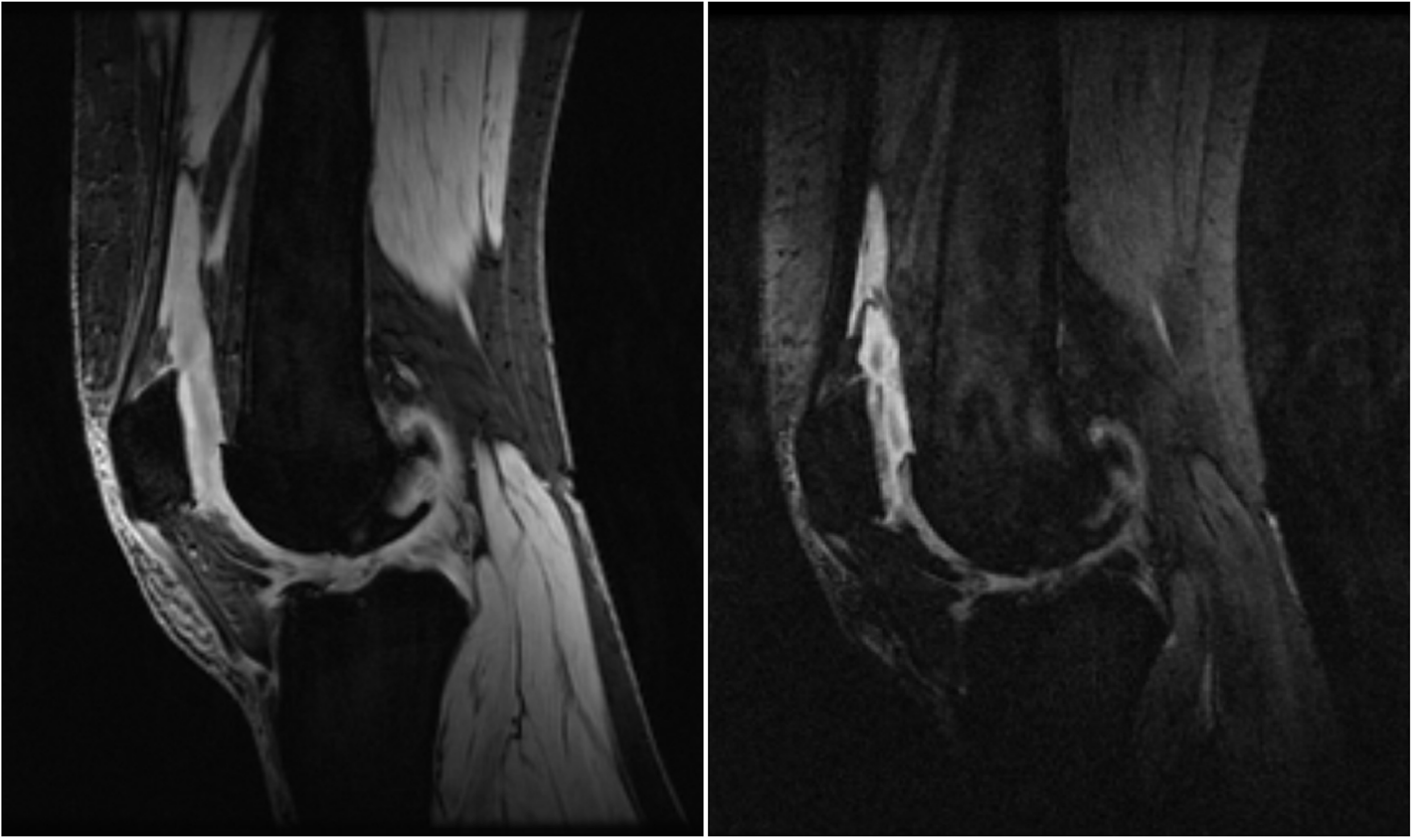
Sagittal qDESS images at the level of the patella, T2 effects dominate the contrast difference between the two echoes S^+^ (left) and S^−^ (right)

qDESS scans were performed directly before CE-MRI, using the sagittal 3D qDESS sequence [18] with TE1 = 9 ms and TE2 = 46.7 ms for echoes before and after the spoiler gradient, respectively, TR = 26.0 ms; matrix size 256 × 256; flip angle = 25°; FOV = 20 cm, a slice thickness of 3 mm, and using water-only excitation. Typically, S^+^ denotes the signal at the first echo, before the spoiler, which mostly has a T1/T2 contrast, while S^−^ denotes the signal at second echo, after the spoiler, which additional T2 and diffusion weighting [18]. The sequence was run with a spoiler gradient of duration 3.4 ms on the slice axis and a gradient area of 15,660 μs*G/cm (156 ms*mT/m), providing strong diffusion weighting. This area corresponds to a gradient inducing a phase difference of 20 cycles over the slice. Scan time was approximately 5 min. This gave a total of two images per slice (Fig. 1).

### Image processing

CE-MR images were evaluated qualitatively according to the synovitis grading, while the qDESS scans required image processing after acquisition. This image processing was performed using custom software (The MathWorks, Natick, MA, USA) created by McWalter et al. [20]. The qDESS images were processed to optimize the contrast between the synovial membrane and synovial fluid. The resulting images were created as a linear combination of the echo 1 (S^+^) and echo 2 (S^−^) images according to the equation:

$$ \mathrm{Synovitis}\ \mathrm{image}={S}^{+}-{\beta S}^{-} $$

where S^+^ and S^−^ are the images on echoes 1 and 2 respectively. The image processing software uses a subtraction ratio, where a coefficient *β* is used to null the synovial fluid accordingly. Simulations were used to determine β that nulled the fluid signal, using the Extended Phase Graph (EPG) model [22] of the qDESS sequence and known values of T_1_ and T_2_ relaxation times and diffusivity for synovial fluid (3620 ms, 767 ms and 2.6 μm^2^/ms, respectively) [19, 23]. We found a ratio of β = 2.49, based on EPG calculation with our scan parameters mentioned earlier. Using this ratio and the equation above, synovitis images were created for each patient.

### Image grading

Synovitis on both CE-MRI and qDESS images was scored by a musculoskeletal radiologist with 16 years of experience in reading clinical and research knee MRI scans (EO) using the semi-quantitative scoring method described by Guermazi et al. [24]. Synovitis was scored at 11 different sites throughout the knee (Table 1), and at each location, the synovial membrane was scored based on the maximal thickness on any slice using the following cut-offs: grade 0 if < 2 mm, grade 1 if 2–4 mm, and grade 2 if > 4 mm. Subsequently, a whole-knee synovitis sum score was calculated by summing the scores of all 11 sites. The diagnosis of synovitis was based on the whole-knee synovitis sum score, as follows: normal or equivocal synovitis (sum score 0–4); mild synovitis (sum score 5–8); moderate synovitis (sum score 9–12); and severe synovitis (sum score ≥ 13). Scoring of qDESS and CE-MRI images was performed independently and in random order, blinded for patient details. Scans were scored on all scan planes, using reformatted images from the 3D sequences.

**Table 1:** Sites scored for synovitis according to Guermazi et al. [24]

1. Medial parapatellar recess 2. Lateral parapatellar recess 3. Suprapatellar 4. Infrapatellar 5. Intercondylar 6. Medial perimeniscal	7. Lateral perimeniscal 8. Adjacent to the anterior cruciate ligaments 9. Adjacent to the posterior cruciate ligaments 10. Baker’s cysts 11. Loose bodies

### Statistical analysis

The correlation between whole-joint synovitis sum scores of qDESS MRI and CE-MRI (reference standard) were determined using Spearman’s rank correlation coefficient. Correlation alone is illustrative; therefore, more exploratory the intraclass correlation coefficient (ICC) was measured for absolute agreement. A correlation coefficient of 0.40–0.59 is considered as moderate, 0.6–0.79 as strong, and 0.8–1 as very strong. Site-specific correlations were also evaluated for all 11 sites separately. Receiver operating characteristic (ROC) analysis was performed to determine the diagnostic performance of the whole-joint synovitis sum score of qDESS MRI, using CE-MRI as the reference standard. Both qDESS and CE-MRI scores were categorized into two categories using the previously published cut-offs [24] and then a tabulation of these two categorized scores was done. Sensitivity, specificity, positive predictive value (PPV), and negative predictive value (NPV) were calculated along with 95% confidence intervals (CI). First, ROC analyses were performed for the diagnosis of synovitis with severity of mild or higher, moderate or higher, and severe using the original cut-off values as described [24]. Finally, the ROC analysis was repeated with adjusted cut-off values of the qDESS whole-joint sum score, based on Youden’s index [25]. A *p* value of < 0.05 was considered statistically significant. Statistical analysis was performed using SPSS (version 25, IBM Corp., Armonk, NY, USA).

## Results

Thirty-one patients (14 females and 17 males; mean age 58 years) were included in this study, of which 6 (19%) had radiographic OA with a severity of K&L grade 1, 10 (32%) had K&L grade 2, 8 (26%) had K&L grade 3, and 7 (23%) had end-stage grade 4 radiographic OA. Baseline characteristics are presented in Table 2.

**Table 2:** Baseline patient characteristics

Sex male (%)	14 (45.2%)
Mean age in years ± SD	57.6 ± 10.0
Mean BMI in kg/m^2^ ± SD	27.5 ± 4.4
Symptomatic knee	Left: *n* = 15 Right: *n* = 16
Radiographic OA severity (K&L grade)	Grade 0: *n* = 0
	Grade 1: *n* = 6
	Grade 2: *n* = 10
	Grade 3: *n* = 8
	Grade 4: *n* = 7

### Imaging findings

**Fig. 2:**
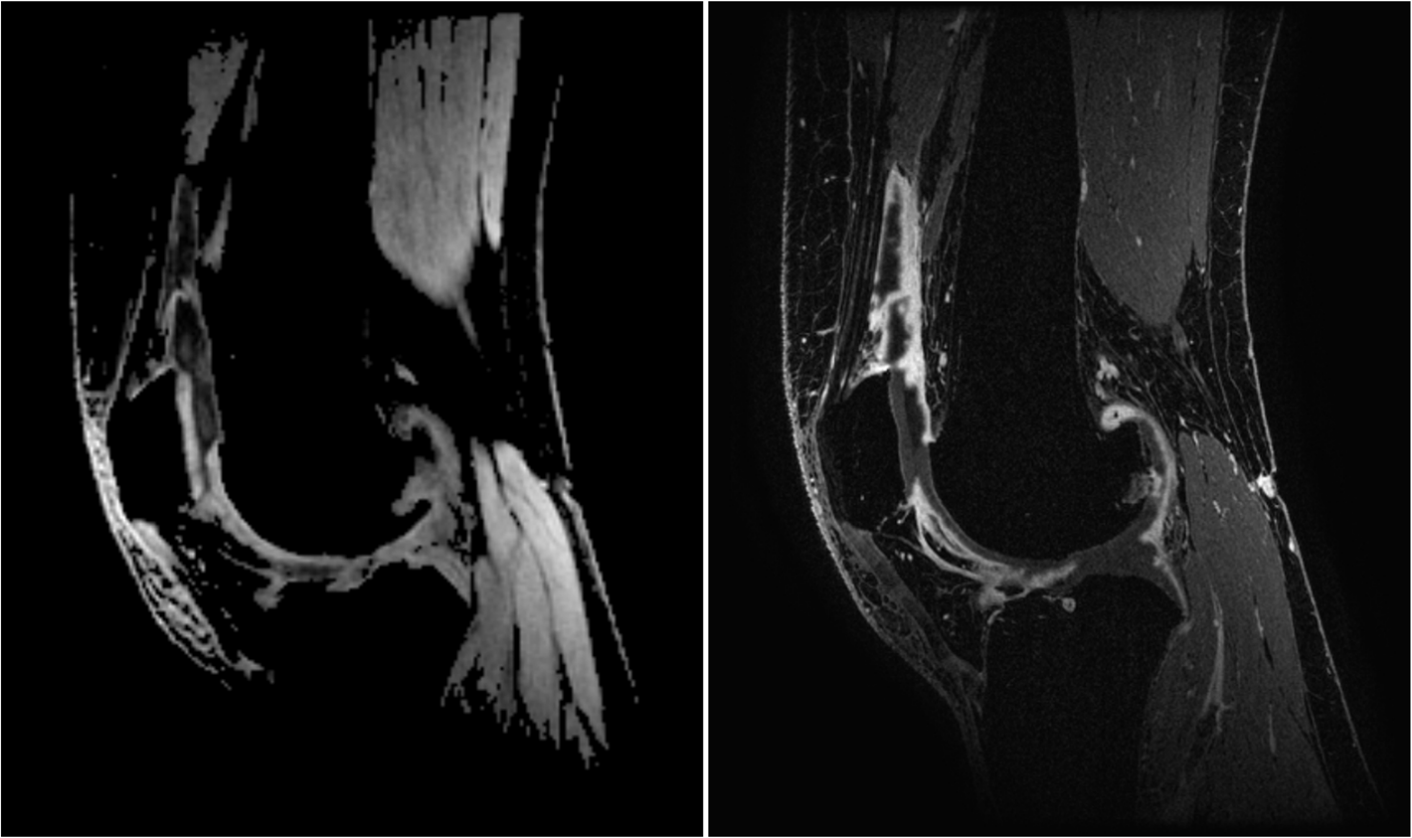
Sagittal qDESS hybrid difference image (left) and CE-MRI (right), both at the level of the patella

**Fig. 3:**
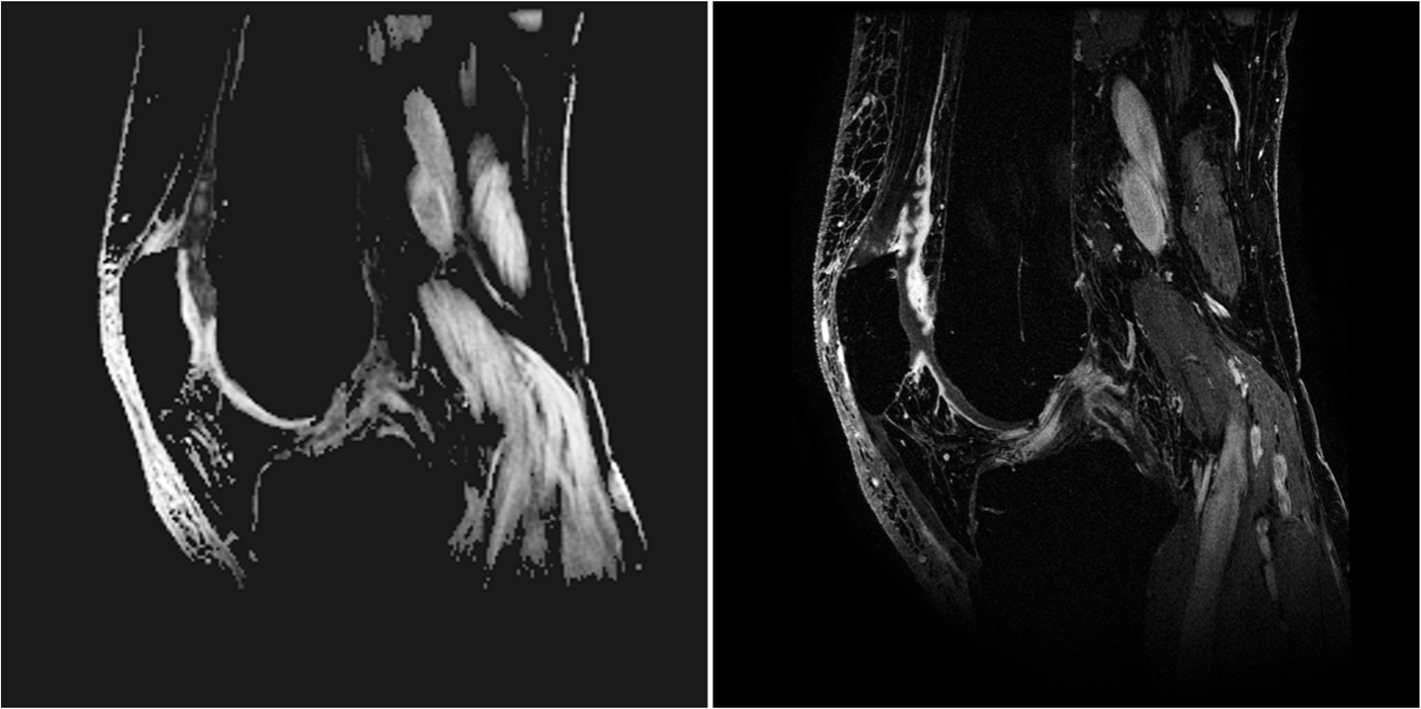
Sagittal qDESS hybrid difference image (left) and CE-MRI (right), both at the level of the origin of anterior cruciate ligament

On CE-MRI, 11 (35.5%) patients had no synovitis, 9 (29.0%) had mild synovitis, 6 (19.4%) had moderate synovitis, and 5 (16.1%) had severe synovitis. On qDESS MRI, 10 out of 31 patients (32.3%) had no synovitis, 13 (41.9%) had mild synovitis, 8 (25.8%) had moderate synovitis, and none had severe synovitis, when the cut-off values of the whole-joint synovitis sum scores were used as defined by Guermazi et al. [24]. qDESS MRI whole-knee sum score showed a mean (SD) of 5.16 (3.75) compared to 7.13 (4.66) for CE-MRI whole-knee. Representative qDESS and CE-MRI images are shown in Figs. 2 and 3.

### Correlation analysis

**Fig. 4:**
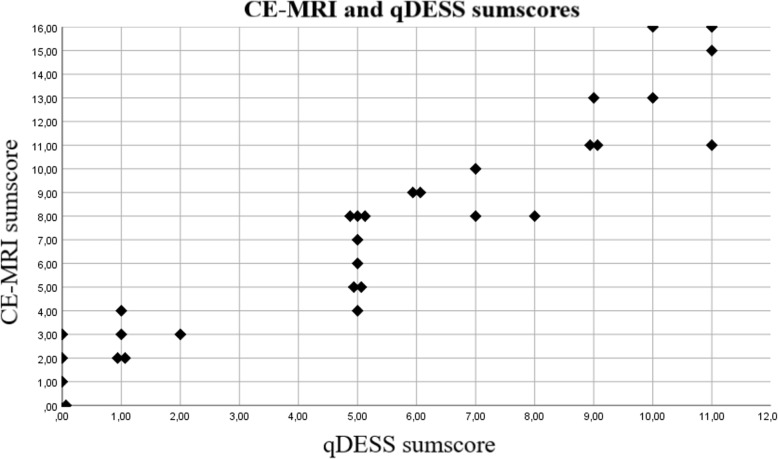
Scatterplot of Guermazi sumscores from both CE-MRI and qDESS

Very strong correlation was found between whole-joint synovitis sum scores of qDESS and CE-MRI (Spearman’s rank correlation coefficient 0.96 (95%CI 0.91–0.98), *p* < 0.001). The scatterplot of all datapoints can be found in Fig. 4. The ICC for absolute agreement was 0.84 (95%CI 0.14–0.95) (Table 3).

**Table 3:** Site-specific correlations

	Spearman’s correlation (*p* value)	ICC absolute agreement (95%CI)
1. Medial parapatellar recess	0.74 (< 0.001)	0.70 (0.45–0.84)
2. Lateral parapatellar recess	0.82 (< 0.001)	0.81 (0.65–0.91)
3. Suprapatellar	0.89 (< 0.001)	0.85 (0.71–0.93)
4. Infrapatellar	0.60 (< 0.001)	0.49 (0.09–0.74)
5. Intercondylar	0.41 (0.022)	0.30 (−0.02–0.57)
6. Medial perimeniscal	0.52 (0.003)	0.44 (0.08–0.69)
7. Lateral perimeniscal	0.67 (< 0.001)	0.58 (0.22–0.78)
8. Adjacent to the anterior cruciate ligaments	0.65 (< 0.001)	0.59 (0.30–0.78)
9. Adjacent to the posterior cruciate ligaments	0.84 (< 0.001)	0.83 (0.68–0.92)
10. Baker’s cysts	0.95 (< 0.001)	0.97 (0.93–0.98)
11. Loose bodies	Not applicable	Not applicable
Whole-joint synovitis sum score	0.96 (< 0.001)	0.84 (0.14–0.95)

When each of the 11 regions was analyzed individually, the highest correlations (> 0.8) were observed for the lateral parapatellar recess, suprapatellar, adjacent to the posterior cruciate ligament, and in Baker’s cyst. Correlation was low for the intercondylar site (Table 3). There were no patients who had synovial thickening around a loose body.

### ROC analysis

The results of the ROC analyses are shown in Table 4. The diagnostic performance of qDESS MRI for detecting mild or higher degree of synovitis showed an AUC (std. error) of 0.98 (0.02), using the original cut-off values, with an accompanying sensitivity and specificity of 1.00 (95%CI 0.80–1.00) and 0.91 (95%CI 0.57–1.00), respectively. For detection of severe synovitis, however, a sensitivity of 0 (95%CI 0–0.537) was found and a specificity of 1.00 (0.84–1.00). After adjusting the cut-off values, the cut-off values changed from 5 to 4, 9 to 6, and 13 to 9, for mild or higher, moderate or higher, and severe synovitis, respectively. Also, the sensitivity and specificity changed after cut-off adjustment, especially for severe synovitis, where the sensitivity increased to 1.00 (95%CI 0.46–1.00) and specificity increased to 0.89 (95%CI 0.69–0.97). The results of the ROC analysis after optimization are shown in Table 5.

**Table 4:** Diagnostic performance of qDESS MRI for mild, moderate, and severe synovitis

Synovitis grade based on CE-MRI	AUC (std. error)	Cut-off value of whole-joint synovitis sum score [24]	TP	TN	FP	FN	Sensitivity (95%CI)	Specificity (95%CI)	PPV (95%CI)	NPV (95%CI)
Mild or higher (*n* = 20)	0.98 (0.02)	≥ 5	20	10	1	0	1.00 (0.80–1.00)	0.91 (0.57–1.00)	0.95 (0.74–1.00)	1.00 (0.66–1.00)
Moderate or higher (*n* = 11)	0.98 (0.02)	≥ 9	8	20	0	3	0.73 (0.39–0.93)	1.00 (0.80–1.00)	1.00 (0.60–1.00)	0.87 (0.65–0.97)
Severe (*n* = 5)	0.96 (0.03)	≥ 13	0	26	0	5	0 (0–0.54)	1.00 (0.84–1.00)	–	0.84 (0.66–0.94)

*AUC* area under the curve, *TP* true positive, *TN* true negative, *FP* false positive, *FN* false negative, *PPV* positive predictive value, *NPV* negative predictive value

**Table 5:** Diagnostic performance of qDESS MRI for mild, moderate, and severe synovitis using adapted cut-offs values of whole-joint synovitis sum score

Synovitis grade based on CE-MRI	AUC (std. error)	Optimized cut-off value of whole-joint synovitis sum score	TP	TN	FP	FN	Sensitivity (95%CI)	Specificity (95%CI)	PPV (95%CI)	NPV (95%CI)
Mild or higher (*n* = 20)	0.98 (0.02)	≥ 4	20	10	1	0	1.00 (0.80–1.00)	0.91 (0.57–1.00)	0.95 (0.74–1.00)	1.00 (0.66–1.00)
Moderate or higher (*n* = 11)	0.98 (0.02)	≥ 6	11	18	2	0	1.00 (0.68–1.00)	0.90 (0.67–0.98)	0.85 (0.54–0.97)	1.00 (0.78–1.00)
Severe (*n* = 5)	0.96 (0.03)	≥ 9	5	23	3	0	1.00 (0.46–1.00)	0.89 (0.69–0.97)	0.63 (0.26–0.90)	1.00 (0.82–1.00)

*AUC* area under the curve, *TP* true positive, *TN* true negative, *FP* false positive, *FN* false negative, *PPV* positive predictive value, *NPV* negative predictive value

## Discussion

Our findings have shown that the qDESS synovitis images can differentiate between the synovial membrane and joint effusion, with high correlation for mild and moderate synovitis. While the contrast between the synovial fluid and membrane for the qDESS synovitis images are visually not as good as the T1-weighted contrast-enhanced sequence images, the synovial membrane is clearly distinguishable. qDESS systematically underestimated synovitis severity compared to CE-MRI. Adjustment of the cut-off values increased the agreement of qDESS, especially for severe synovitis.

In this study, we included patients with knee OA ranging from K&L 1–4, whereas in a previous pilot study [20] data of patients with knee OA K&L 2 or 3 was analyzed. We believe that, because of its non-contrast properties, DESS ultimately holds promise as an (early) OA imaging biomarker that can be applied routinely in clinical patient care and research. It can be implemented widely in existing MRI protocols, and become a useful addition to the multi-tissue capability of MRI for OA assessment. The inclusion of a technique capable of visualizing synovitis in MRI protocols may facilitate identification of patients with an “inflammatory” OA phenotype who may benefit from targeted anti-inflammatory treatment.

Lower synovial scores were found using qDESS than using CE-MRI. A possible explanation for this could be that diffusion parameters measured by qDESS on the edges of synovial tissue are almost equal to synovial fluid, which makes the synovial tissue look smaller on qDESS than on CE-MRI. Further optimization of the qDESS technique, both with regard to the acquisition and image processing may in future reduce the systematic underestimation of synovitis severity.

There are other non-CE-MRI scoring methods, such as WORMS [26], KOSS [27], BLOKS [28], and MOAKS [29], that do not require a contrast agent. However, all these methods score synovitis indirectly based on a combination of both effusion and synovial hypertrophy.

Diffusion tensor imaging (DTI) is another technique that can image synovitis in knee OA non-invasively, without using a contrast agent. It is used to study the structure of biological tissue. The idea of using DTI for knee synovitis is based on previous experience in brain imaging, where high fractional anisotropy (FA) is positively correlated with pro-inflammatory cytokines. Agarwal et al. [30] found that the synovium showed higher FA values compared to surrounding tissue. Double inversion recovery (DIR) MRI is another method, which enables the evaluation of inflamed synovium by simultaneously suppressing fat signal and water signal intensity of the joint effusion [31–33]. Also, a recent study showed that using fluid attenuation inversion recovery (FLAIR) MRI, by nullifying the fluid signal, inflamed synovium was detectable without using a contrast agent [34]. Ultrasound is an alternative imaging modality to assess synovitis. However, although ultrasound may be particularly useful to diagnose synovitis, it has limitations with regard to quantitative assessment. Also, while MRI allows the evaluation of all potential locations of synovitis in the knee joint, both superficial and deep, ultrasound can only visualize superficial areas.

The strengths of our study are that we included patients with all severities of radiographic OA (K&L grade 1 to 4) and that we were able to perform different MRI sequences, including contrast-enhanced MRI in as many as 31 patients. Thirty-one patients can also be seen as a low amount; however in this study, it is enough as it is mostly exploratory. There are certain other limitations to our study. First, the data presented in this manuscript is cross-sectional; therefore, no link regarding disease progression could be made. Second, to create the qDESS synovitis images, some minor post processing is required. However, we believe that these are technical issues which can be addressed relatively easily, and we expect that the demonstration of good diagnostic performance by this and other studies may accelerate the translation of the adapted qDESS sequence and post-processing algorithms. No histology was assessed in this study; however, we think that arthroscopic biopsy is not the best reference method because the most important thing we want to know in this study is the load of the inflammation, which cannot be assessed using biopsy. Another limitation is that the scan time of qDESS sequence is around 5 min. The acquisition takes this long, due to the multiple echoes that are required for the diffusivity, and also the very large FOV used in this study played a role. However, this version of qDESS can also be used to assess the T2 relaxation times and apparent diffusion coefficient of cartilage [18, 19]. However, as there is no need for a contrast agent, total examination time is shorter than CE-MRI. Finally, we did not externally validate our results in an independent cohort, which we consider an essential next step in the evaluation of qDESS in follow-up research. As a further consideration, the optimal unenhanced MRI technique to depict synovitis is not yet known and future research should continue to investigate the different unenhanced MRI techniques and compare with qDESS MRI.

## Conclusion

In conclusion, synovitis detection is possible without the need for an intravenous contrast agent by using hybrid images created using qDESS MRI. Redefinition of cut-off values is needed for this scoring, because qDESS consistently shows slight underdetection compared to CE-MRI.

## Data Availability

The datasets used and/or analyzed during the current study are available from the corresponding author on reasonable request.

## Abbreviations

AUC: Area under the curve
CE: Contrast enhanced
CI: Confidence interval
DIR: Double inversion recovery
DTI: Diffusion tensor imaging
EPG: Extended phase graph
FA: Fractional anisotropy
FLAIR: Fluid attenuation inversion recovery
GFR: Glomerular filtration rate
K&L: Kellgren and Lawrence
MRI: Magnetic resonance imaging
NPV: Negative predictive value
OA: Osteoarthritis
PPV: Positive predictive value
qDESS: Quantitative double-echo in steady-state
ROC: Receiver operating characteristic
SPGR: Spoiled gradient-echo sequence
VAS: Visual analog scale
